# Bleeding rectal polyp as an atypical presentation of intestinal schistosomiasis: A case report from Egypt

**DOI:** 10.1371/journal.pntd.0013972

**Published:** 2026-02-09

**Authors:** Mohamed M. Elhoseeny, Ahmed Sallam, Ahmed E. Eladl, Amira A. A. Othman

**Affiliations:** 1 Internal Medicine Department, Faculty of Medicine, Suez University, Suez, Egypt; 2 Hepatology and Gastroenterology Department, Mahalla Hepatology Teaching Hospital, El Mahalla El Kobra, El Gharbeya, Egypt; 3 Pathology Department, Faculty of Medicine, Mansoura University, Mansoura, Egypt; Advanced Centre for Chronic and Rare Diseases, INDIA

## Abstract

**Background:**

Schistosomiasis remains an important public health challenge in Egypt despite decades of control programs. Intestinal involvement is relatively common but usually presents with diffuse mucosal disease, ulcerations, or multiple polyps. A solitary bleeding rectal polyp as the sole manifestation is exceedingly rare and can be mistaken for inflammatory bowel disease or colorectal neoplasia. This report aims to highlight this diagnostic challenge within the Egyptian context.

**Case Presentation:**

A 28-year-old Egyptian male from a rural area presented with a 3-month history of intermittent lower abdominal pain and rectal bleeding. Physical examination was unremarkable except for mild lower abdominal tenderness and blood on digital rectal exam. Laboratory tests, including inflammatory markers, were normal; notably, stool microscopy was repeatedly negative for schistosome ova, despite the presence of RBCs and WBCs. Colonoscopy identified a solitary, pedunculated, and ulcerated rectal polyp (1.5 × 3.0 cm) at 20 cm from the anal verge, which was completely resected. Histopathological examination confirmed the diagnosis by demonstrating viable *Schistosoma mansoni* ova within eosinophilic granulomas and, critically, an adult worm residing in the submucosal vasculature, confirming active infection.The patient achieved full clinical recovery after praziquantel therapy, and this case underscores the importance of integrating parasitological, endoscopic, and histopathological perspectives when managing atypical colorectal lesions in endemic regions.

**Conclusion:**

This case is a striking example of intestinal schistosomiasis masquerading as a sporadic colorectal neoplasm. In endemic regions like Egypt, schistosomiasis must be considered in the differential diagnosis of solitary rectal polyps, even with negative stool examinations. The definitive diagnosis hinges on histopathological analysis, which is indispensable for guiding correct management and avoiding unnecessary interventions. This report reinforces the ongoing, evolving challenge of schistosomiasis in Egypt post-control programs.

## Author summary

Schistosomiasis remains a health problem in Egypt, even after years of control programs. While intestinal schistosomiasis often causes widespread inflammation, we report a very unusual case where it appeared as a single bleeding polyp in the rectum, looking just like a tumor or inflammatory bowel disease. This 28-year-old man had rectal bleeding and abdominal pain, but standard stool tests failed to detect the parasite. Only after removing the polyp during colonoscopy and examining it under the microscope was the diagnosis clear: the tissue contained both schistosome eggs and an adult worm. This case teaches us that in regions where schistosomiasis still occurs, doctors must consider this infection even when tests are negative and the presentation is misleading. Pathological examination is key to correct diagnosis and avoiding unnecessary treatments. Our report emphasizes that as classic schistosomiasis becomes rarer, unusual forms may become more common, requiring updated clinical awareness.

## Introduction

Schistosomiasis is one of the most prevalent parasitic diseases worldwide, with an estimated 230–250 million people requiring preventive treatment annually, particularly in Africa, the Middle East, and South America, according to recent WHO reports [1]. In Egypt, *Schistosoma mansoni* and *Schistosoma haematobium* have historically been highly endemic, with widespread distribution along the Nile Delta and Valley [2]. Over the past five decades, national control programs using praziquantel mass drug administration and snail control have significantly reduced prevalence, but schistosomiasis persists, especially in rural communities [3]. This shifting epidemiology means that classic presentations are becoming less common, while atypical ones may be overlooked. Although national control programs have markedly reduced schistosomiasis transmission in Egypt, persistent foci remain. Historical localized data from the Nile Delta show high baseline village-level prevalence of ~36–70%, demonstrating sustained transmission in specific rural settings. Elmorshedy et al. (2020) highlight these focal patterns in high- versus low-prevalence areas of the Nile Delta [4]. Recent elimination efforts, including intensified surveillance and school-based surveys conducted in Upper Egypt between 2016 and 2017, found overall urinary schistosomiasis prevalence as low as ~1.3% among schoolchildren, though district-level prevalence varied widely (0–13.4%) [5]. This focal persistence, against a backdrop of national control success, contributes to an evolving epidemiology where classic presentations are becoming less common, while sporadic and atypical cases, such as the one reported here, may be overlooked. Together, these data support that schistosomiasis persists at low countrywide prevalence but with focal pockets of transmission, particularly in rural riverine communities.

The clinical spectrum of schistosomiasis is broad. The urinary form, usually due to *S. haematobium*, leads to hematuria, bladder wall changes, and long-term risk of squamous cell carcinoma [6]. Hepatosplenic schistosomiasis, mainly caused by *S. mansoni*, results in periportal fibrosis, portal hypertension, and variceal bleeding [7]. Intestinal schistosomiasis, though common, is often under-recognized because its clinical manifestations are frequently mild or nonspecific and may overlap with other gastrointestinal conditions, particularly in chronic or low-intensity infections. Patients may present with chronic diarrhea, abdominal pain, tenesmus, or occult gastrointestinal bleeding [8]. Endoscopic findings include mucosal congestion, petechiae, ulcerations, pseudopolyps, or strictures [9].

Although early autopsy studies reported rectosigmoid polyps in 17–20% of patients with long-standing infection [10], polyp formation is now considered an uncommon clinical presentation in the modern endoscopic era. The marked decline in rectosigmoid polyp formation in the modern era is likely attributable to widespread praziquantel-based mass treatment and improved control programs, which shorten the duration of infection, reduce cumulative egg deposition within the intestinal wall, and limit the chronic granulomatous and fibrotic responses necessary for polyp development [11]. The pathogenesis involves a chronic granulomatous reaction to eggs trapped in the mucosa and submucosa, leading to localized fibrosis and mucosal proliferation [12]. Histopathological and clinical series have shown that schistosomal polyps are typically multiple and diffuse, reflecting widespread egg deposition within the intestinal wall, whereas solitary, large, bleeding rectal polyps are rarely reported [12,13]. Few cases have been documented in the international literature, and even fewer from Egypt in the modern post–post-mass-treatment era.

While intestinal schistosomiasis remains a public health concern in rural Egypt, its clinical presentation has evolved post-mass-treatment, with classic symptomatic cases declining and atypical manifestations becoming proportionally more significant. A solitary, bleeding rectal polyp as the sole manifestation represents one such atypical presentation. Critically, definitive histological confirmation of active infection, particularly the demonstration of an adult worm within the lesion, is rarely documented in reported cases. We present a detailed case of a young Egyptian male with a solitary bleeding rectal polyp as the initial and sole manifestation of intestinal schistosomiasis. This case is notable for its close endoscopic resemblance to a neoplastic polyp and the definitive histological proof of active infection (both ova and adult worm) despite non-contributory standard parasitological tests. This report aims to highlight this unusual presentation, discuss the diagnostic challenges it poses within the Egyptian healthcare context, and emphasize the critical role of histopathology in ensuring correct patient management. Unlike previous case reports, which primarily described ova within polyps, our case is among the very few to document both viable ova and an adult worm embedded in the submucosa of a solitary rectal polyp. This dual finding provides a rare, diagnostically definitive demonstration of active infection and highlights a diagnostic pitfall of direct clinical relevance in post-control Egypt.

## Methods

### Study design and ethical approval

This is a retrospective, single-patient case report documenting a rare clinical presentation of intestinal schistosomiasis. The case was managed at the gastroenterology clinic of Mahalla Hepatology Teaching Hospital (El Mahalla El Kobra, Egypt), with collaborative input from faculty members of the Internal Medicine Department at Suez University Faculty of Medicine (Suez, Egypt) and the Pathology Department at Mansoura University Faculty of Medicine (Mansoura, Egypt). Institutional policy at Suez University Faculty of Medicine confirms that single-patient case reports documenting standard clinical care do not require formal ethical review board approval, provided patient confidentiality is maintained and informed consent is obtained.

### Patient consent and confidentiality

Written informed consent was obtained from the patient for all diagnostic and therapeutic procedures, including colonoscopy, polypectomy, and histopathological examination. Additional written informed consent was obtained for the publication of this case report and all accompanying clinical and pathological images. All patient identifiers have been removed to protect confidentiality.

### Data collection and clinical evaluation

Clinical data were extracted retrospectively from the patient’s electronic medical record. The diagnostic evaluation followed a standardized institutional protocol for the assessment of lower gastrointestinal bleeding and abdominal pain. This included a detailed clinical history, comprehensive physical examination, and systematic laboratory investigation. Laboratory workup comprised a complete blood count with differential, measurement of inflammatory markers (erythrocyte sedimentation rate and C‑reactive protein), and quantification of fecal calprotectin. Hepatic and renal function panels were performed. Parasitological assessment involved stool microscopy on three consecutive samples. Abdominal ultrasonography was conducted to evaluate hepatosplenic morphology and rule out signs of portal hypertension.

### Endoscopic procedure and sample collection

Colonoscopy was performed by a board-certified gastroenterologist (A.S.) using a high-definition video colonoscope (Olympus CF-HQ190L) after a split-dose polyethylene glycol bowel preparation, following current guidelines for optimal bowel cleansing. The identified solitary rectal polyp was characterized according to the Paris classification. It was resected en bloc using hot snare polypectomy with blended electrosurgical current to ensure complete excision and adequate histological assessment. Immediate hemostasis was achieved by deploying two endoclips at the resection base. The resected specimen was retrieved, measured, and oriented before fixation in 10% neutral buffered formalin for 24 hours. It was then submitted in its entirety for histopathological processing [14].

### Histopathological analysis

The formalin-fixed polyp specimen was routinely processed through dehydration, clearing, and paraffin embedding. Serial sections were cut at 4-μm thickness using a rotary microtome and mounted on glass slides. Sections were stained with hematoxylin and eosin (H&E) according to standard histological protocols. All slides were examined by a consultant gastrointestinal pathologist (A.E.E.) using a light microscope (Olympus BX43) under bright-field illumination at magnifications ranging from 40× to 400 × . The diagnosis of intestinal schistosomiasis was established based on the identification of characteristic viable *S. mansoni* ova, distinguished by their lateral spines and surrounding eosinophilic granulomatous reaction, along with the visualization of an adult worm within a submucosal venule. The absence of epithelial dysplasia, malignancy, and histological features indicative of inflammatory bowel disease, such as crypt architectural distortion, basal lymphoplasmacytosis, or neutrophilic cryptitis unrelated to granulomas, was systematically assessed and confirmed [15].

### Treatment and follow-up

Following histopathological confirmation, the patient was treated according to national schistosomiasis management guidelines with a single oral dose of praziquantel (40 mg/kg). He received counseling on avoiding freshwater exposure. Clinical follow-up was scheduled at 6 weeks and 6 months, with assessment of symptoms and repeat stool microscopy.

### Data analysis and reporting

As a descriptive case report, formal statistical analysis was not performed. Data are presented narratively with clinicopathological correlation. Figures were prepared from original endoscopic and histopathological images using Adobe Photoshop CC 2024 for cropping and labeling only, without alteration of diagnostic content.

## Results

### Patient information

A 28-year-old Egyptian male, working as a building guard, presented to the gastroenterology clinic with a 3-month history of intermittent crampy lower abdominal pain and occasional passage of fresh blood per rectum. The bleeding was moderate in amount, occurring with or shortly after defecation, without mucus or melena. There was no change in stool frequency or consistency apart from occasional loose stools. He denied anorexia, significant weight loss, fever, or night sweats.

The patient was a chronic smoker and reported occasional recreational drug use but no alcohol intake. He lived in a rural community with intermittent exposure to freshwater canals, which constitutes a recognized risk factor for *S. mansoni* transmission in this region. He had no past medical or surgical history and no family history of colorectal cancer or inflammatory bowel disease.

### Clinical findings

On examination, he was of average build (BMI ~ 23 kg/m²), with stable vital signs. There was mild tenderness in the lower abdomen, without rebound, guarding, or palpable mass. The liver edge was not palpable, the spleen was not enlarged, and there were no peripheral stigmata of chronic liver disease. Digital rectal examination revealed no palpable lesion, but blood was noted on the glove.

### Diagnostic assessment

#### Laboratory investigations.

Initial laboratory evaluation revealed a hemoglobin level of 13.2 g/dL, total leukocyte count of 6.8 × 10⁹/L with a differential of 60% neutrophils, 30% lymphocytes, 6% monocytes, and 4% eosinophils, and a platelet count of 230 × 10⁹/L. This borderline eosinophilia (4%, within the upper-normal range), while consistent with a parasitic etiology, was considered non-specific and insufficient to confirm the diagnosis in the absence of other supportive findings. Inflammatory markers showed an ESR of 18 mm/h and a C-Reactive Protein (CRP) of 3 mg/L. Liver and renal function tests were within normal limits.

Fecal calprotectin was obtained due to the initial consideration of inflammatory bowel disease in a young patient with rectal bleeding. The result was mildly elevated at 103 µg/g (reference <50 µg/g), supporting the presence of low-grade mucosal inflammation but remaining non-specific for its etiology.

Stool microscopy performed on three consecutive samples demonstrated numerous red blood cells (50–55 per high-power field [HPF]) and white blood cells (12–15/HPF), but no parasitic ova were detected.

Given the patient’s age and clinical presentation, initial differentials included sporadic adenomatous polyp, early inflammatory bowel disease, and, less likely, colorectal malignancy. Infective etiologies were considered less probable due to repeatedly negative stool studies, underscoring the diagnostic challenge. More sensitive diagnostic modalities, such as serological assays or polymerase chain reaction (PCR) for schistosomiasis, were not available in our clinical setting and were therefore not performed. Given the initial higher suspicion for a neoplastic or inflammatory polyp and the decision to proceed with a diagnostic–therapeutic colonoscopy, external referral for specialized parasitological testing was not pursued, as histopathology was expected to provide a definitive diagnosis.

#### Imaging.

Abdominal ultrasound demonstrated mildly increased hepatic echogenicity, a nonspecific finding that can be associated with hepatic steatosis or early inflammatory changes. However, the normal spleen size, absence of ascites, and normal portal vein diameter provided no sonographic evidence of periportal fibrosis or portal hypertension characteristic of hepatosplenic schistosomiasis. In view of the persistent rectal bleeding, a colonoscopy was subsequently performed following standard polyethylene glycol split-dose preparation.

#### Endoscopic findings.

**Fig 1:**
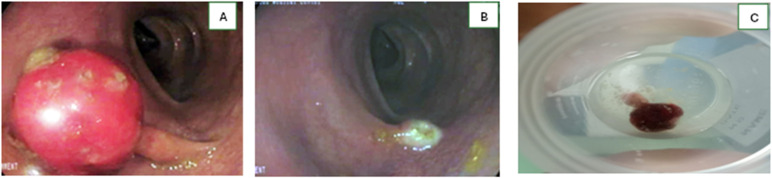
Endoscopic view of the solitary rectal polyp. **(A)** A pedunculated polyp (Paris classification 0-Ip) with an ulcerated and friable surface is seen in the proximal rectum. The stalk is clearly visible. **(B)** the post-polypectomy site, **(C)** Totally removed pedunculated polyp.

Colonoscopy showed normal mucosa from the cecum to the sigmoid colon. In the proximal rectum, 20 cm from the anal verge (corresponding to the rectosigmoid junction region), there was a pedunculated polyp measuring 1.5 × 3.0 cm, with an ulcerated, friable surface (Paris classification 0-Ip, indicating a pedunculated polyp). The surrounding mucosa appeared mildly erythematous but otherwise normal. No additional polyps or mucosal abnormalities were observed (Fig 1).

After the written informed consent was obtained, endoscopic polypectomy was performed using a hot snare technique. The polyp was successfully resected en bloc with adequate margins. The resection site was carefully inspected, and hemostasis was secured using endoclips prophylactically. The procedure was uneventful, and the patient recovered well. The specimen was sent for histopathological examination in its entirety.

#### Histopathology.

**Fig 2:**
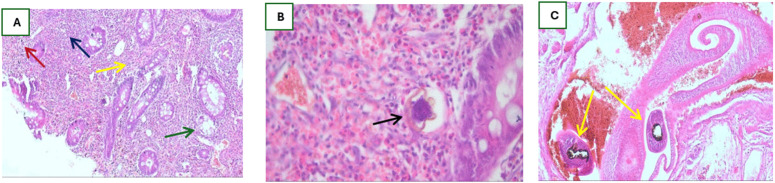
Histopathological features of intestinal schistosomiasis (H&E). (A) Photomicrograph showing colonic mucosa with granulomatous inflammation surrounding *S. mansoni* eggs (hematoxylin and eosin stain, × 100). The granulomas consist of epithelioid histiocytes, lymphocytes, and eosinophils. Multiple distorted and hyperplastic colonic glands are noted, with associated mucosal fibrosis and chronic inflammatory cell infiltration, consistent with chronic schistosomal colitis. Arrows indicate key lesions: Schistosoma egg (red), surrounding granulomatous inflammation (blue), distorted colonic glands (green), and fibrotic changes (yellow). The findings are consistent with chronic schistosomal colitis. (B) High-power view of intestinal schistosomiasis (H&E, × 400).

Microscopic examination (H&E) at low power revealed mucosal ulceration with dense inflammatory infiltrates extending into the submucosa. Multiple viable *S. mansoni* ova, characterized by their distinctive lateral spines, were embedded within the mucosa and submucosa. These ova were surrounded by granulomatous inflammation rich in eosinophils, lymphocytes, and multinucleated giant cells, with associated areas of fibrosis. High-power examination further confirmed the presence of a viable ovum with a prominent surrounding eosinophilic reaction. Most notably, sections demonstrated an adult schistosome worm (representing one member of a worm pair); this presence does not necessarily imply a unisex infection, but rather reflects histological sampling of a single worm within a submucosal venous channel, providing direct evidence of active infection. No dysplasia or malignancy was identified (Fig 2).

The simultaneous demonstration of viable ova and an adult worm within the same lesion not only confirmed active infection but also eliminated alternative diagnoses such as neoplastic or purely inflammatory polyps with secondary parasitic colonization. Critically, the absence of histopathological features essential for IBD, such as crypt architectural distortion, basal plasmacytosis, or cryptitis and crypt abscesses unrelated to granulomas, effectively excluded this diagnosis.

A well-formed granuloma is seen surrounding an *S. mansoni* egg with a characteristic lateral spine (center). The granulomatous reaction is composed predominantly of epithelioid histiocytes, lymphocytes, and numerous eosinophils. Adjacent colonic glands show chronic inflammatory infiltration and reactive epithelial changes. **(C):** Adult Schistosoma worm in a submucosal venous vascular space (Yellow arrows) (H&E, × 200). Histopathology demonstrates a cross-section of an adult Schistosoma worm within a dilated submucosal vein. The worm is identified by its eosinophilic tegument and internal structures, surrounded by numerous erythrocytes within the vascular lumen. The adjacent mucosa shows chronic inflammatory cell infiltration and early fibrotic reaction, characteristic of intestinal schistosomiasis.

### Therapeutic Intervention and Follow-Up

Following histological confirmation, the patient was treated with praziquantel at 40 mg/kg as a single oral dose, in accordance with national guidelines [16]. He was counseled to avoid freshwater exposure and to adhere to follow-up.

At the 6-week follow-up, the patient reported resolution of abdominal pain and rectal bleeding. At 6 months, he remained asymptomatic. A follow-up stool examination was negative for ova. Repeat colonoscopy was advised at 12 months, but was not performed as the patient was lost to long-term follow-up.

## Discussion

This case report aimed to document a rare solitary bleeding rectal polyp as the initial manifestation of intestinal schistosomiasis in post-control Egypt and to highlight the diagnostic challenges it poses. Polypoid lesions in schistosomiasis are rare but clinically important because they may mimic adenomatous polyps, colorectal carcinoma, or inflammatory bowel disease [17]. Several reports from endemic countries have described similar cases. Issa et al. [18] reported a young woman with a colonic polyp containing S. mansoni ova despite negative stool examinations. Emara et al. [3] described five Egyptian cases of colonic schistosomiasis mimicking cancer, polyps, or IBD, highlighting the diagnostic pitfalls. In Saudi Arabia, Al-Zubaidi et al. reported a case of large rectal polyps secondary to Schistosoma infection [19]. In Nigeria, David et al. [20] and Alyhari et al. [21] documented huge colonic granulomas of schistosomiasis mimicking cancer. Unlike these prior reports, the present case is distinguished by the histological demonstration of both viable ova and an adult worm within a solitary polyp, providing definitive evidence of active local infection and offering a clear pathophysiological illustration of polyp formation and ulceration.

This case vividly illustrates an evolving clinical challenge in post-control schistosomiasis endemic regions like Egypt. As the overall prevalence decreases, the classic clinical presentations taught in textbooks become less common for the average clinician to encounter [3]. This leads to a natural “acumen shift,” where the index of suspicion for schistosomiasis lowers. Consequently, a solitary, ulcerated polyp in a young adult is far more likely to be presumed to be a sporadic adenoma or a manifestation of inflammatory bowel disease than a parasitic infection. Our case underscores that schistosomiasis remains a “great mimicker” and must be retained in the differential diagnosis of colorectal polyps, especially in patients with epidemiological exposure, even in an era of perceived low transmission. Failure to do so risks misdiagnosis, unnecessary interventions, and a missed opportunity to treat the underlying systemic infection.

A recent review emphasized that schistosomal polyps can occur anywhere in the colon, most frequently in the rectosigmoid region [22]. They may be solitary or multiple, sessile or pedunculated, and vary in size from a few millimeters to several centimeters. Bleeding and anemia are common symptoms, but occasionally they are incidental findings [23].

Our case is notable for being a solitary, ulcerated, and bleeding rectal polyp in a young Egyptian male with negative stool microscopy results. This underscores the limitations of stool examination, which has reduced sensitivity in light infections or in chronic cases with fewer viable eggs [24]. The patient’s borderline eosinophil count (4%) further supported a parasitic etiology, though its upper-normal value underscored the non-specific nature of this finding. Colonoscopy with histopathology remains the gold standard for diagnosis in such cases.

Polyp formation in intestinal schistosomiasis results from chronic deposition of eggs in the intestinal wall, which triggers a host immune response characterized by granulomatous inflammation, fibroblast proliferation, and mucosal hyperplasia [25]. Over time, this localized reaction produces a polypoid lesion, while ulceration may occur secondary to vascular obstruction or chronic inflammatory activity [26]. In our case, the presence of an adult worm within a submucosal venous channel of the polyp itself represents a highly specific finding that elegantly illustrates the local pathophysiology. Continuous local deposition of eggs likely acted as a nidus for the sustained granulomatous response driving polyp growth. Moreover, the worm’s occupation of a submucosal vessel, together with the mass effect of the surrounding granuloma, may have contributed to localized vascular compromise, ischemia, and eventual ulceration of the overlying mucosa, directly explaining the patient’s presenting symptom of rectal bleeding. The entrapment of an adult worm within a submucosal venule, as opposed to its typical residence in the mesenteric vasculature, could result from localized migration and impaired venous drainage caused by the developing granulomatous reaction and fibrotic changes within the polyp itself. The solitary nature of the polyp in this case, rather than the more typical diffuse polyposis reported in chronic schistosomiasis, can be explained by the confined presence of an adult worm pair, likely a worm pair, continuously depositing eggs into a localized segment of the rectosigmoid vasculature.

The diagnostic challenges in such presentations are multifaceted. In the current era of reduced schistosomiasis prevalence in Egypt, clinicians may have a lower index of suspicion, allowing this “great mimicker” to be mistaken for more common conditions. This is compounded by the fact that in chronic or light-intensity infections, which are increasingly common in post-control settings, egg output can be minimal and focal. This can occur in cases of chronic infection, low worm burden, single-sex infections, or when eggs are trapped within tissue granulomas. In the specific context of a solitary polyp, eggs are sequestered within the granulomatous tissue and may not be released into the fecal stream in detectable numbers, leading to repeatedly negative stool microscopy despite active local infection, as exemplified by our case. Stool microscopy, although widely used, has limited sensitivity in chronic or light infections due to intermittent egg excretion [24,27]; thus, a negative stool examination, as in our patient, cannot exclude the disease. The mildly elevated fecal calprotectin level was interpreted as reflecting the localized mucosal inflammation from the intense granulomatous reaction to the schistosome ova, rather than a diffuse inflammatory bowel disease. The mild increase in hepatic echogenicity on ultrasound was a nonspecific finding, and the absence of sonographic features of periportal fibrosis or portal hypertension ruled out classic hepatosplenic involvement.

Endoscopically, ulcerated polyps caused by schistosomiasis may closely resemble adenomatous or malignant polyps, while associated mucosal inflammation and pseudopolyps can overlap with inflammatory bowel disease, particularly ulcerative colitis [28,29]. Therefore, in endemic or post-control regions, schistosomiasis should be considered in the differential diagnosis of solitary colorectal polyps—especially in young patients without typical risk factors for neoplasia—even when stool microscopy is negative. In our case, the solitary ulcerated pedunculated polyp was endoscopically indistinguishable from an adenoma, underscoring the importance of histological confirmation. This case informs clinical decision-making by highlighting that when schistosomiasis is suspected despite negative parasitology, endoscopic resection with histopathological examination is both diagnostic and therapeutic. Following histological confirmation, empiric praziquantel therapy should be administered to treat any residual infection. The mild surrounding mucosal erythema likely represented a localized inflammatory response to the polyp itself, rather than evidence of a diffuse colitidic process such as inflammatory bowel disease. Furthermore, acute infectious colitis was considered unlikely given the patient’s lack of diarrhea, fever, or systemic symptoms, alongside the endoscopic finding of a solitary polyp rather than diffuse mucosal inflammation.

Inflammatory bowel disease (IBD) was definitively excluded based on the absence of characteristic histopathological features, including crypt architectural distortion, basal plasmacytosis, or chronic active colitis patterns unrelated to granulomas.

The patient’s occasional recreational drug use was evaluated as a potential confounder; however, the absence of systemic symptoms, normal inflammatory markers, and lack of histopathological evidence of drug-induced ischemia or vasculitis made this an unlikely contributor to the lesion.

Based on this experience, we propose refined diagnostic considerations for young patients from endemic regions presenting with colorectal polyps. While colonoscopy with polypectomy and histology remains the cornerstone for both diagnosis and therapy, clinicians should maintain a high pre-test suspicion for schistosomiasis. Where available, more sensitive non-invasive tests, such as serological assays or circulating antigen detection, may provide critical diagnostic clues before endoscopy, especially in cases with negative stool microscopy [24,27]. A positive result in these tests would appropriately raise suspicion, guide preoperative discussions, and ensure timely preparation for praziquantel therapy pending histological confirmation.

The implications of this case extend beyond the individual patient. For clinicians in endemic regions, schistosomiasis should remain a key differential diagnosis when evaluating colorectal polyps, particularly in young patients without classical risk factors for neoplasia. In low-prevalence, post-control settings, atypical presentations can undermine screening efforts that rely on classic symptoms or positive stool microscopy. Therefore, heightened clinical suspicion and readiness to use histopathology are essential. Histopathological examination of all resected polyps is essential, with careful search for schistosome ova regardless of stool microscopy results or endoscopic appearance, to avoid misdiagnosis. Even when a lesion is completely excised, praziquantel therapy should still be administered to eradicate residual infection and prevent recurrence [16]. From a public health perspective, despite the success of national control programs, focal transmission persists in Egypt, especially in rural Nile Delta regions, leading to morbidity that may mimic neoplasia or inflammatory bowel disease and consume substantial diagnostic resources [3]. Such cases argue for integrating atypical schistosomiasis presentations into medical curricula and continuous professional development, ensuring that clinicians in transitioning endemic regions maintain diagnostic vigilance. Greater awareness among gastroenterologists and pathologists is therefore critical to prevent unnecessary surgery or IBD therapy and to ensure timely, cost-effective antiparasitic treatment.

The strength of this case lies in its clear and comprehensive documentation of an unusual presentation of schistosomiasis in Egypt, with clinicopathological correlation supported by histopathology demonstrating both viable ova and an adult worm, a finding that is diagnostically definitive for active infection. This provides a complete diagnostic picture that underscores the value of histological confirmation in endemic settings. Notwithstanding its instructive value, this report has certain limitations. The absence of long-term endoscopic surveillance precludes definitive confirmation of complete mucosal healing. Furthermore, without serological or circulating antigen tests, we cannot definitively determine whether this represented an early infection or a chronic low-burden infection. However, the patient’s sustained symptomatic resolution and negative follow-up stool examination at six months provide robust clinical evidence of successful infection clearance. Furthermore, the definitive diagnosis relied on histopathology without adjunctive molecular confirmation (e.g., PCR or circulating antigen detection). While the histological demonstration of both an adult worm and viable ova is diagnostically definitive and considered diagnostic, these serological or molecular tools could have provided additional pre-endoscopic clues and quantified the parasite burden. Their integration in future studies, where available, could further enhance the diagnostic workup for such atypical presentations.

The principal novelty of this case lies in its stark challenge to common clinical assumptions regarding intestinal schistosomiasis in a modern Egyptian context. The combined histological finding of both viable ova and an adult worm within a solitary rectal polyp is rarely documented in the recent case literature [13], providing a diagnostically definitive and pathophysiologically illustrative example of active infection. First, it demonstrates that a solitary, bleeding polyp can be the sole and initial manifestation of the disease, moving beyond the classic textbook description of diffuse mucosal involvement or multiple pseudopolyps. Second, it underscores a critical diagnostic blind spot: the profound inadequacy of stool microscopy, which repeatedly failed to detect the infection, nearly leading to a misdiagnosis of neoplasia or IBD. Finally, the histological documentation of both viable ova and an adult worm within the submucosal vasculature is a rare and diagnostically definitive finding that provides incontrovertible evidence of active local infection and elegantly illustrates the underlying pathophysiology. This triad, an atypical solitary lesion, deceptive negative parasitology, and definitive histological proof, elevates this case from a simple clinical anecdote to a salient teaching point with significant implications for diagnostic reasoning in endemic areas.

This case highlights a broader implication: as endemic regions transition through post-control phases, atypical, deceptive manifestations of schistosomiasis will become proportionally more common. Medical curricula and continuing education should therefore integrate such atypical scenarios, ensuring younger physicians, who may not have firsthand experience with classic schistosomiasis, retain a high index of suspicion.

## Conclusion

This case highlights an atypical presentation of intestinal schistosomiasis as a solitary bleeding rectal polyp, closely mimicking neoplasia or inflammatory bowel disease. In Egypt’s evolving epidemiological landscape, where classic forms are waning, clinicians must remain alert to its potential for atypical and misleading presentations. Negative stool examinations do not exclude the diagnosis, reflecting the limitations of parasitological testing in chronic or light infections. Colonoscopy with meticulous histopathological evaluation remains the diagnostic cornerstone. Ultimately, this case underscores the need for heightened awareness among gastroenterologists and pathologists in endemic regions, both to prevent misdiagnosis and unnecessary interventions and to ensure timely, curative antiparasitic therapy that improves patient outcomes.

Future studies combining endoscopic, histological, and molecular diagnostic tools are needed to define the true burden of atypical schistosomiasis presentations and to guide evidence-based surveillance strategies in endemic regions.

### Declarations

#### Ethics approval and consent to participate:.

- Institutional policy confirms that single-patient case reports do not require IRB approval.
- Written informed consent was obtained for all diagnostic and therapeutic procedures.
- Patient management followed established clinical protocols in accordance with the Declaration of Helsinki.

#### Consent for publication.

- Written informed consent was obtained from the patient for publication of this case report and any accompanying images.

**Note:** All authors read and approved the final manuscript.
